# Primary Fibular Osteomyelitis in Children: A Systematic Review

**DOI:** 10.7759/cureus.41345

**Published:** 2023-07-04

**Authors:** Nia Nikkhahmanesh, Neeraj Vij, Ashish S Ranade, Mohan Belthur

**Affiliations:** 1 Orthopedics, University of Arizona College of Medicine, Phoenix, USA; 2 Orthopedics, Blooming Buds Centre for Pediatric Orthopaedics, Deenanath Mangeshkar Hospital and Research Centre, Pune, IND; 3 Pediatric Orthopedics, Phoenix Children’s Hospital, University of Arizona College of Medicine, Phoenix, USA

**Keywords:** hematogenous, acute, pediatric, osteomyelitis, fibula

## Abstract

Osteomyelitis of the fibula is rare and is especially rare in children. The published literature is limited to case series and is thus lacking a comprehensive description of the disease. The purpose of this systematic review is to provide the first comprehensive summary of the demographics, presenting symptoms, laboratory values, microbiology, and treatment results of osteomyelitis of the fibula in children based on the existing literature. This institutional review board (IRB)-exempt systematic review followed the Preferred Reporting Items for Systematic Reviews and Meta-Analyses Protocol (PRISMA-P) guidelines. Three search engines were used for a total of 239 studies. Twenty-six studies were screened by full text. Twelve articles underwent a quantitative analysis. Due to limited data and heterogenous reporting, the data were summarized descriptively. The methodologic quality of the studies was evaluated based on the Newcastle-Ottawa scale. The average age was 7.71±3.49 years, and males comprised 57% of the 21 cases. The most common presenting symptoms were fever (86%), antalgic gait (57%), and localized tenderness (81%). The most common site of involvement was the distal third of the fibula (90%). The average C-reactive protein (CRP) was 90.1±38.3 mg/L, and the average erythrocyte sedimentation rate (ESR) was 58.8±21.2 mm/hour. *Staphylococcus aureus *was the most cultured pathogen reported in 10/21 cases (48%). Open surgery was performed in 17/21 cases (81%), and there were no reported complications. Fever, antalgic gait, and localized tenderness should raise the index of suspicion. Prompt laboratory and radiographic evaluations can help reduce delays in diagnosis and improve outcomes. Blood and tissue cultures are currently performed in about half of the cases. Improvement in our microbiologic diagnosis has the potential to improve antibiotic selection. Local methicillin-resistant *Staphylococcus aureus* (MRSA) prevalence must be taken into consideration when starting empiric antibiotic treatment. Surgical treatment is often required with a low complication rate. The clinical and laboratory parameters identified in this study have the potential for integration into a composite clinical score.

## Introduction and background

Osteomyelitis can be characterized by chronicity, source, and microbiology [1]. Osteomyelitis with an onset of <4 weeks before presentation is referred to as acute, between four and eight weeks as subacute, and >8 weeks as chronic. The most common mechanism of infection is hematogenous inoculation secondary to an episode of bacteremia [1]. Other possible sources include contiguous spread or direct inoculation. Microbiologic sources include bacteria, fungi, or mycobacteria.

*Staphylococcus aureus* is the most frequently encountered pathogen in acute pediatric osteomyelitis, with an incidence of 70%-90%, and tends to manifest through the vascular spread of the pathogen from a distant primary site of infection. Other reported bacterial pathogens include *Staphylococcus epidermidis*, *Streptococcus* spp., *Pseudomonas*, *Haemophilus influenzae*, coagulase-negative *Staphylococcus*, and *Corynebacterium* spp. [2].

Osteomyelitis of the fibula is considered rare, and there is scant literature describing its disease course and treatment. This is especially true in the pediatric population [3]. The purpose of this systematic review is to summarize the epidemiology, presenting symptoms, laboratory values, microbiology, and treatment results of osteomyelitis of the fibula in children.

## Review

Materials and methods

The Preferred Reporting Items for Systematic Reviews and Meta-Analyses Protocol (PRISMA-P) guidelines were strictly followed. Additionally, the review was registered with PROSPERO (CRD42021281293).

Search Strategy

The literature search was performed by the authors using the following searches: Medical Subject Heading (MeSH) term: population, search fields: children, pediatric, and child; MeSH term: diagnosis, search fields: osteomyelitis, fibula, and infection. PubMed, Scopus, Embase, and Ovid/Medical Literature Analysis and Retrieval System Online (MEDLINE) databases were used. Search fields were alternated until no new articles were revealed. At that point, the search was considered complete.

Study Screening and Selection

**Figure 1:**
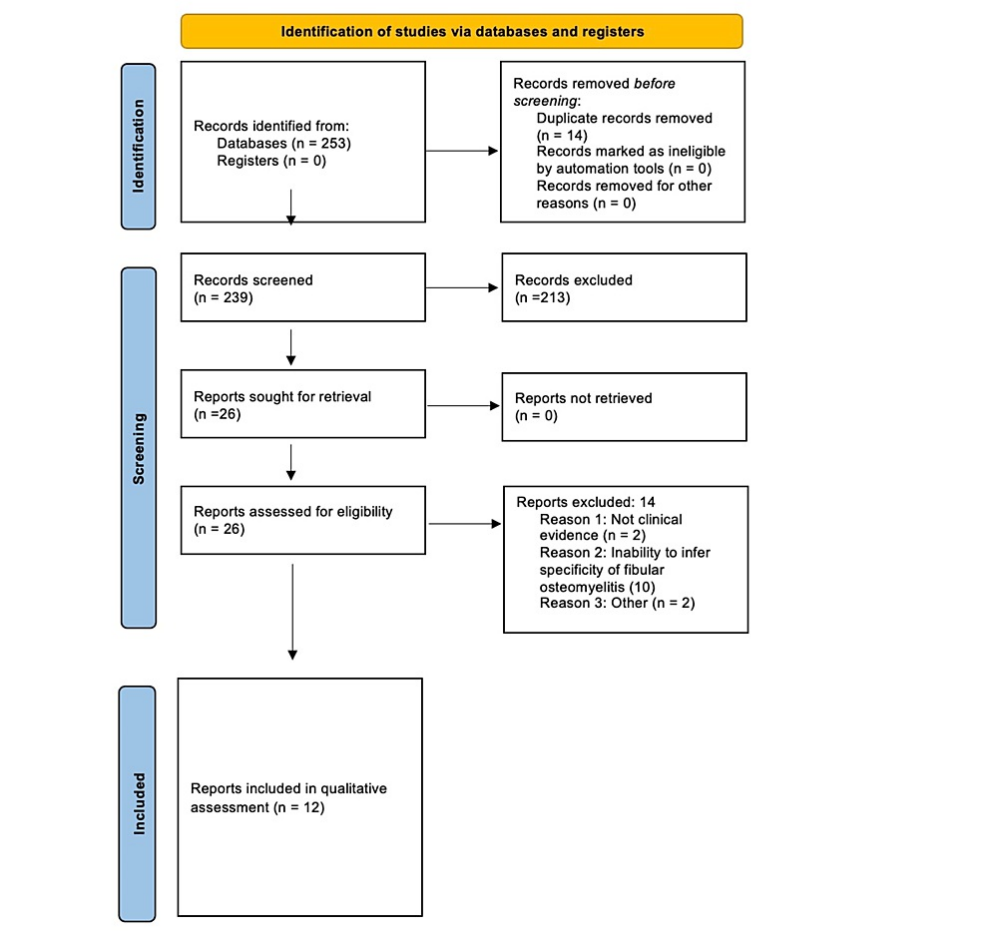
Our PRISMA 2022 flow diagram depicting our study selection process. PRISMA: Preferred Reporting Items for Systematic Reviews and Meta-Analyses

The search yielded a total of 253 studies (Figure 1). After the removal of duplicates, 239 articles remained. Based on the title and abstract review performed by all four of the authors, a total of 26 articles were included for qualitative review. The bibliographies of all articles reviewed were thoroughly evaluated to determine if any other relevant articles had been unintentionally overlooked. The independent full-text screenings of the articles were performed by all four authors. Of the 26 screened articles, 14 articles did not meet our inclusion/exclusion criteria (Table 1). The resulting 12 articles underwent quantitative analysis.

**Table 1:** Our inclusion and exclusion criteria as applied independently by three of the authors during the title/abstract review and full-text screening.

Inclusion Criteria	Exclusion Criteria
Age of <18	Level V evidence
The diagnosis of acute hematogenous osteomyelitis of the fibula	The absence of at least one data table beyond the demographics
Level IV evidence or higher	Minimum follow-up of 12 months

Data Collection

The categories for data gathering included population demographics, presenting features, laboratory values, imaging modalities, the extent of fibular involvement, microbiologic diagnosis, medical management, surgical management, and complications. The variables included under demographic features were age and gender. The presenting features were recorded as binary variables. The C-reactive protein (CRP), erythrocyte sedimentation rate (ESR), leukocyte count, and initial temperature were recorded. The use and positive results of ultrasound (US), computed tomography (CT), and MRI were recorded. The involvement of the proximal, middle, or distal third of the fibula and extension to the proximal tibia-fibula joint or distal intraarticular extension was recorded. The use and positive results of aspirate, surgical, and blood cultures were recorded. The disease course and the hospital course were recorded. The duration and choice of oral and intravenous antibiotics were recorded. The type and number of surgeries (open versus US- or CT-guided percutaneous drainage) were recorded. Post-operative complications including septicemia, knee stiffness, ankle stiffness, ankle septic arthritis, amputation, and mortality were recorded.

Quality Assessment

To assess the included studies, guidelines from the revised assessment of multiple systematic reviews (R-AMSTAR) were followed [4]. Since all the published literature is limited to a level of clinical evidence of I, the modified Newcastle-Ottawa scale was chosen as the appropriate risk of bias tool [5]. This tool has been modified to appropriately evaluate the methodologic quality of case reports and case series in light of their most common pitfalls [5]. It includes a series of eight questions that fall within the domains of selection, ascertainment, causality, and reporting [6]. This resulted in eight studies with scores of 7/8 and four studies with scores of 6/8 (Table 2).

**Table 2:** The results of the application of the modified Newcastle-Ottawa scale* to our 12 studies. *As proposed by Murad et al. [5]

Study	Selection	Ascertainment	Causality	Reporting
Bayram et al., 2019 [2]	*	**	***	*
Begovic et al., 2020 [6]	*	**	***	*
Borjian et al., 2012 [7]	*	**	***	*
Griebel et al., 1985 [8]	*	**	**	*
Henrikson et al., 1974 [9]	*	**	**	*
Huang et al., 2013 [10]	*	**	**	*
Jlalia and Kaffel, 2020 [11]	*	**	***	*
Kaziz et al., 2020 [12]	*	**	**	*
Nadau et al., 2020 [13]	*	**	***	*
O’Neill et al., 2003 [14]	*	**	***	*
Percin et al., 1993 [15]	*	**	***	*
Singh et al., 2017 [16]	*	**	***	*

Data Abstraction and Statistical Analysis

Due to heterogeneous data, a meta-analysis could not be performed. All data were summarized descriptively according to the recommendations of Dhawan et al. [4]. Frequencies and proportions were calculated for categorical variables. Means and standard deviation were calculated for continuous variables. Data abstraction and figure creation were performed with Microsoft Excel version 16.42 (Microsoft® Corp., Redmond, WA).

Results

Demographic Features

Our review included 12 articles [2,6-16] for a total of 21 pediatric patients with osteomyelitis of the fibula (Table 3). The average age at the time of diagnosis was 7.71±3.48 years. Males comprised 12/21 cases (57%), and females comprised 9/21 (43%).

**Table 3:** Article information and patient demographic data.

Study	Country	Year	Journal	Total Patients	Age	Percentage of Male Involvement	Fibula Location
Bayram et al. [2]	Turkey	2019	JBJS Case Connector	1	4 years	100%	Distal third
Begovic et al. [6]	Serbia	2020	Jundishapur Journal of Microbiology	1	13 years	0%	Proximal third
Borjian et al. [7]	Iran	2012	Journal of Orthopaedics and Traumatology	1	14 years	100%	Middle and distal third
Griebel et al. [8]	USA	1985	Journal of Pediatric Orthopaedics	1	7 years	0%	Distal third
Henrikson et al. [9]	Sweden	1974	Journal of Pediatric Surgery	1	8 months	0%	Distal third with distal intraarticular extension
Huang et al. [10]	China	2013	BMC Infectious Diseases	1	11 years	100%	Distal third
Jlalia and Kaffel [11]	Tunisia	2020	The Pan African Medical Journal	1	10 years	100%	Middle third
Kaziz et al. [12]	Tunisia	2020	Archives de Pédiatrie	7	7.71 years	71.4%	All distal third
Nadau et al. [13]	France	2020	Archives de Pédiatrie	4	8.2 years	50%	All distal third
O’Neill et al. [14]	USA	2003	Journal of the Arkansas Medical Society	1	30 months	0%	Distal third
Percin et al. [15]	Turkey	1993	Acta Orthopaedica Belgica	1	5 years	0%	Distal third
Singh et al. [16]	India	2017	International Journal of Orthopaedics Sciences	1	8 years	100%	Middle and distal third

Presenting Symptoms

The presence of fever was reported in 18/21 cases (86%), antalgic gait in 12/21 cases (57%), localized tenderness in 17/21 cases (81%), erythema in 10/21 cases (48%), and edema in 14/21 cases (67%). Other reported symptoms included chills in 3/21 cases (14%), while stupor, skin rash, vomiting, and discharge were each only reported in 1/21 cases (5%).

Laboratory Assessment

Laboratory testing involved measuring CRP, ESR, leukocyte count (WBC), wound cultures, and blood cultures (Table 4). CRP was reported in 15/21 cases (71%) with an average of 90.1±38.3 mg/L. Initial ESR was reported in 15/21 cases (71%) with an average of 58.8±21.2 mm/hour. Initial WBC was reported in 19/21 cases (90.5%) with an average of 12,399±3,130 cells per microliter. Additionally, fever was reported in 16/21 cases (76%) with an average temperature of 38.8±0.58 degree Celsius.

**Table 4:** The mean laboratory values of inflammatory markers and the temperature of the 21 patients evaluated. NR, not reported; CRP, C-reactive protein; ESR, erythrocyte sedimentation rate

Study	Mean CRP (mg/L)	Mean ESR (mm/hour)	Mean WBC (Cells/mL)	Mean Temperature (Degree Celsius)
Bayram et al., 2019 [2]	20.2	30	18,910	NR
Begovic et al., 2020 [6]	101.7	90	6,800	40
Borjian et al., 2012 [7]	NR	119	12,000	38
Griebel et al., 1985 [8]	NR	53	10,000	39
Henrikson et al., 1974 [9]	NR	28	NR	NR
Huang et al., 2013 [10]	NR	NR	8,600	NR
Jlalia and Kaffel, 2020 [11]	20	50	17,470	38.2
Kaziz et al., 2020 [12]	103	56.6	12,571	39.2
Nadau et al., 2020 [13]	111	NR	10,800	>38.5
O’Neill et al., 2003 [14]	NR	NR	NR	NR
Percin et al., 1993 [15]	NR	56	18,600	37.6
Singh et al., 2017 [16]	40	60	12,000	NR

Imaging

Ultrasound imaging was utilized in 7/21 cases (33%), while MRI was used in only 1/21 cases (5%). No reports of CT imaging were made. All seven reports of US imaging reported positive findings, and the one case reporting MRI usage also reported positive findings.

Extent of Fibular Involvement

The most common site of involvement was the distal third of the fibula in 19/21 cases (90%) followed by the middle third of the fibula in 3/21 cases (14%). Only one report of osteomyelitis of the proximal third of the fibula was mentioned along with only one report of distal intraarticular extension. No cases reported contiguous spread into the proximal tibia-fibula joint.

Microbiology

*Staphylococcus aureus* was the most cultured pathogen reported in 10/21 cases (48%). Of the 10 cases, four cases were unspecified *S. aureus*, while the other six cases were reported as methicillin-sensitive *Staphylococcus aureus* (MSSA). Among the cultured pathogens were group A beta-hemolytic *Streptococcus*, *Neisseria meningitidis*, and *Mycobacterium tuberculosis*, to name a few.

Disease Course

The mean time from symptom onset to diagnosis was 10.3±16.5 days. The mean hospital length of stay was 17.7±7.54 days.

Medical Management

The mean duration of intravenous antibiotic therapy was 20.3±9.86 days (Table 5). The mean duration of oral antibiotic therapy was 23.5±3.55 days. The mean duration of both intravenous and oral antibiotic therapy was 35.5±6.24 days. Only 4/21 cases (19%) were fully resolved with medical management alone.

**Table 5:** Oral and intravenous antibiotic choice and the mean duration of treatment and pathogen targeted. NR, not reported; PAS, 4-aminosalicylic acid; INH, isoniazid; MSSA, methicillin-sensitive *Staphylococcus aureus*; IV, intravenous

Study	Oral Antibiotic Choice	Mean Oral Antibiotic Duration (Days)	IV Antibiotic Choice	Mean IV Antibiotic Duration (Days)	Mean Total Antibiotic Duration	Pathogen
Bayram et al., 2019 [2]	NR	NR	Vancomycin+cefazolin	42	42	C. striatum
Begovic et al., 2020 [6]	Ciprofloxacin	21	Ceftriaxone	21	42	E. coli
Borjian et al., 2012 [7]	Cloxacillin	NR	NR	NR	NR	S. aureus
Griebel et al., 1985 [8]	Cefazolin	NR	Penicillin G	NR	NR	Group A beta-hemolytic *Streptococcus*
Henrikson et al., 1974 [9]	Penicillin, streptomycin, PAS, and INH	NR	NR	NR	NR	M. tuberculosis
Huang et al., 2013 [10]	Voriconazole	168	NR	NR	168	Histoplasma capsulatum
Jlalia and Kaffel, 2020 [11]	NR	21	NR	NR	21	NR
Kaziz et al., 2020 [12]	Oxacillin-gentamycin	25	Oxacillin-gentamycin	14	33.2	MSSA, *Haemophilus influenzae*, and *Salmonella* typhi
Nadau et al., 2020 [13]	Rifampicin, fusidic acid, and Augmentin	NR	Cefazolin, gentamicin, cefotaxime, fosfomycin, and oxacillin	NR	NR	S. aureus
O’Neill et al., 2003 [14]	Cefuroxime	14	Clindamycin, cefotaxime, and cefuroxime	33	47	Kingella kingae
Percin et al., 1993 [15]	NR	NR	Penicillin G and ceftazidime	35	35	N. meningitidis
Singh et al., 2017 [16]	Linezolid	28	Cefazolin	14	42	MSSA

Surgical Management and Complications

Open surgery was performed in 17 cases (81%). No patients underwent US- or CT-guided percutaneous drainage. There were no reported post-operative complications.

Discussion

This was an institutional review board (IRB)-exempt systematic review that included 12 studies for a total of 21 patients. The average age was 7.71±3.49 years, and males comprised 57% of the 21 cases. The most common presenting symptoms were fever (86%), localized tenderness (81%), and antalgic gait (57%). The most common site of involvement was the distal third of the fibula (90%). The average C-reactive protein (CRP) was 90.1±38.3 mg/L, and the average erythrocyte sedimentation rate (ESR) was 58.8±21.2 mm/hour. *Staphylococcus aureus* was the most cultured pathogen reported in 10/21 cases (48%). Only 19% of patients responded well to medical management alone with 17/21 (81%) necessitating some form of operative treatment. There were no surgical complications.

The nonspecific presentation of pediatric infectious conditions often makes diagnosis difficult [13]. The results of our study may help assist in the initial clinical suspicion and thus facilitate earlier diagnosis and appropriate treatment. Fever, localized tenderness, and antalgic gait were the most reported symptoms among the patients. This is congruent with Nadau et al.’s study, which demonstrated a greater predictive value of foot and ankle osteomyelitis in children for fever in conjunction with localized tenderness [13].

In this review, both ESR and CRP levels were measured in 71% of cases, and WBC was measured in 90.5%. CRP has great utility as both a diagnostic and a monitoring agent [17]. ESR rises and drops slower than CRP levels in acute infections and is no longer considered a routine measurement for diagnosing osteomyelitis [18]. Measuring both ESR and CRP may slightly improve sensitivity and negative predictive value for diagnosis; however, further investigation is necessary to establish a certain diagnostic role in osteomyelitis [18].

Ultrasound was utilized in 7/21 cases, and MRI was only used for 1/21 cases in this study. The utility of imaging studies such as radiography, MRI, CT, and US has been described in the diagnosis; however, there is limited evidence in support of their necessity for the diagnosis and treatment of acute osteomyelitis [19]. The results of our study support this statement with only 38% of the patients pursuing some form of advanced imaging.

The most common organism cultured in our review was *Staphylococcus aureus* with 60% of these cases specifying MSSA and the other 40% of cases remaining unspecified. This is in concordance with published literature [20]. An increase in methicillin-resistant *S. aureus* (MRSA) has been reported in the pediatric population and should always be considered when deciding on empiric antimicrobial treatment [20]. Group A *Streptococcus* was noted in one case in this review and is a common cause of osteomyelitis in the pediatric population between the ages of two and five years [20]. *Haemophilus influenzae* was cultured in only one case, which is consistent with the drastic decrease in the cases of osteomyelitis secondary to *H. influenzae* after the introduction of large-scale vaccinations [20]. Lastly, *Kingella kingae* was reported in one case and is another frequent pathogen encountered in osteoarticular infections in children less than four years of age [20]. However, the results of our review demonstrate that our microbiologic diagnostic capabilities are currently used sparingly. This may represent the high costs associated with these technologies [18,21].

Currently, the treatment of pediatric osteomyelitis involves using empiric antimicrobial therapy. The presence of community-acquired methicillin-resistant *S. aureus* (CA-MRSA) must be considered given the local susceptibility data and patient history [22]. The culture and identification of the offending pathogen allow for definitive therapy with a narrow spectrum representing the cost-effective and responsible use of antibiotic resources [17]. However, the results of our study demonstrate that an improvement in the microbiologic diagnostic capability is required before microbe-specific antibiotic treatment can be initiated [18].

The factors associated with negative outcomes in pediatric osteomyelitis patients include young age, repeated negative cultures, delayed surgery, rural residence, and male sex [17]. No complications were noted in any of the 21 cases represented in this study. However, higher-level clinical evidence with outcome data is required on the topic.

This study is not without its limitations. Due to the rarity of osteomyelitis of the fibula in the pediatric population, this systematic review was limited to case series and is thus of level IV evidence. Due to heterogeneous data and variable follow-up, we are limited in the interpretation of our complication data. With that being said, the inclusion criteria of a minimum of 12-month follow-up was chosen to minimize this risk. As a review of published data, this does introduce a selection bias; however, the broad use of search engines was performed to minimize this risk. The limitation of published literature resulted in a small sample of patients, which does limit the generalizability of our study.

## Conclusions

Pediatric fibular osteomyelitis is rare. Fever, antalgic gait, and localized tenderness should raise the index of suspicion. Prompt laboratory and radiographic evaluation can help reduce the delay in diagnosis and improve outcomes. Blood and tissue cultures are currently performed in about half of the cases. Improvement in our microbiologic diagnosis has the potential to improve antibiotic selection. Local MRSA prevalence must be taken into consideration when starting empiric antibiotic treatment. Surgical treatment is often required with a low complication rate. The clinical and laboratory parameters identified in this study have the potential for integration into a composite clinical score.
